# Uterocutaneous Fistula after a C-Section in a Patient with Second Trimester Fetal Demise and Chorioamnionitis

**DOI:** 10.1155/2021/3255188

**Published:** 2021-09-10

**Authors:** Déborah Wernly, Valérie Besse, Daniela Huber

**Affiliations:** ^1^Department of Obstetrics and Gynecology, Sion's Hospital, Valais, Switzerland; ^2^Department of Radiology, Sion's Hospital, Valais, Switzerland; ^3^Department of Pediatrics, Obstetrics and Gynecology, Geneva University Hospitals, Switzerland

## Abstract

Uterocutaneous fistulae are very rare entities with only about 120 cases reported in the literature. They are mostly described after a C-section or other pelvic surgery. We hereby describe a uterocutaneous fistula in a 41-year-old patient 5 months after a C-section because of a chorioamnionitis and a 22-week fetal demise. One month after the C-section, she underwent a diagnostic hysteroscopy to exclude postoperative intrauterine adhesions. Afterwards, she complained of pelvic pain, persistent metrorrhagia, and significant weight loss during 2 months. She consulted the emergency unit several times, and lastly endometritis was diagnosed. She was treated with antibiotic therapy for 7 days, without significant clinical improvement. She presented at our institution 48 hours after a carbuncle had appeared in her right iliac fossa. A uterocutaneous fistula was diagnosed on the CT scan. The patient received IV antibiotic therapy and underwent a total hysterectomy with bilateral salpingectomy by laparotomy, as she did not want a conservative surgery. The clinical postoperative evolution was favorable. Symptoms of UCF can be very unspecific. To avoid medical wandering and improve the patient's care, UCF should be in the differential diagnostic of abdominal pain after a pelvic surgery. Moreover, in patients with previous C-section and infectious perioperative status, the risk of PID or pelvic abscess must be careful evaluated before intrauterine diagnostic or therapeutic procedures.

## 1. Introduction

Uterocutaneous fistulae (UCF) are abnormal communications between the endometrium and the skin. Around 20 cases were reported in the literature in the last 10 years [1]. The majority of these cases are complications of a C-section, although some are the result of uterine perforation after curettage for a septic abortion [2–4]. Rarely, UCFs are caused by other surgeries such as polymyomectomy by laparotomy [5] or by other mechanisms such as necrobiosis of an intramural fibroid following a C-section [6]. UCF can also appear after a hysteroscopy, performed after a septic event.

The only pathognomonic symptom is the menstrual bleeding through the abdominal scar. Other symptoms are often atypical and may lead to diagnostic delay. An early diagnostic allows a conservative management of the fistulae: medical treatment with antibiotics and hormonal therapy or conservative surgery with the excision of the fistulous tract. As the majority of UCF appear after a C-section, young patients are concerned. Conservative management is therefore crucial if family planning is incomplete.

## 2. Case Presentation

In September 2019, a 41-year-old nulliparous patient had undergone a C-section by midline laparotomy with a corporeal hysterotomy at 22 weeks, in the context of fetal demise and chorioamnionitis. She underwent oral antibiotic therapy with metronidazole for 7 days postoperatively. In October 2019, a diagnostic hysteroscopy was conducted and revealed a posterior uterine myoma and no intrauterine adhesions.

Subsequently, the patient complained of abdominal pain, which led to multiple gynecological consultations. In December 2019, she underwent oral antibiotic therapy with metronidazole and co-amoxicillin for 7 days for suspicion of endometritis. The patient felt no clinical improvement and consulted twice again for abdominal pain and metrorrhagia.

**Figure 1:**
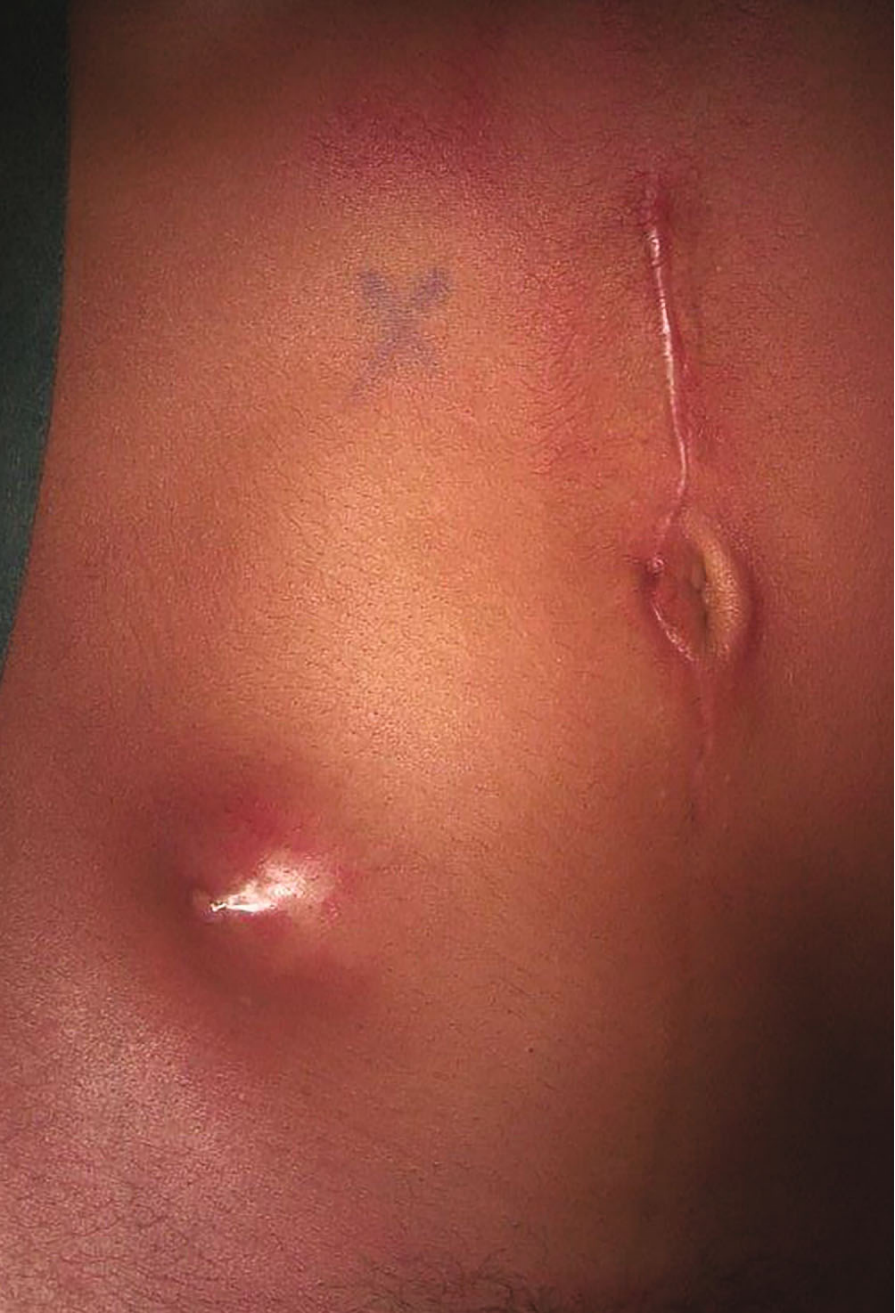
Carbuncle in the right iliac fossa.

In early February 2020, after 4 months of persistent abdominal pain, she presented in our institution because a carbuncle in her right iliac fossa had appeared (Figure 1). She also reported an increased abdominal pain, metrorrhagia, and purulent vaginal discharge. She complained of inappetence and an involuntary 8 kg weight loss during the last 2 months.

**Figure 2:**
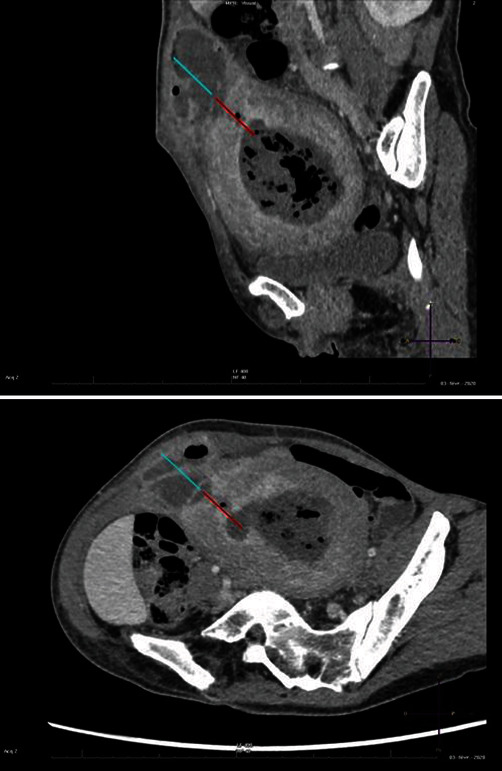
Sagittal and axial MPR reconstruction. Portal phase after iodine contrast injection. The blue arrow shows UCF. The red arrow shows uterine wall defect.

She was hemodynamically stable and had no fever. Laboratory findings showed leukocytosis (13.7 G/L), elevated c-reactive protein (244 mg/L), and anemia (hemoglobin 87 g/L). An abdominal CT scan identified a uterocutaneous fistula with a 7.5 × 7.5 × 11 cm intrauterine collection draining from the right antero-lateral uterine fundus to the abdominal wall (Figure 2).

**Figure 3:**
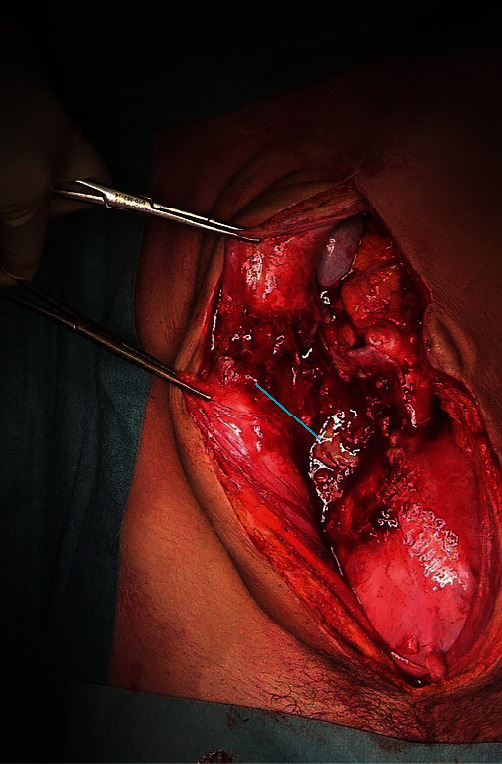
The blue arrow shows fistulae's tract (from the right uterine fundus to the skin) after its excision, before hysterectomy. The green star shows the uterus.

The patient received IV antibiotic therapy. As she was in favor of a radical treatment, a total hysterectomy with bilateral salpingectomy by midline laparotomy was performed.  Figure 3 shows the excision of the fistula tract right before the hysterectomy. The bacteriological analyses identified an *Enterococcus faecalis* strain that was sensitive to co-amoxicillin. The antibiotic therapy was pursued for a total of 14 days with a favorable clinical evolution.

The patient was discharged 9 days after surgery. Two months after, she was treated conservatively for bartholinitis. In May 2020, she was evaluated by our general surgeon for a possible right abdominal wall reinforcement. The results of a CT scan showed no need of further surgery.

## 3. Discussion

UCFs are very rare fistulae with only 120 cases described in the literature over the past 200 years [7]. Complications of a C-section or curettage for septic abortion associated with uterine perforation account for most cases [2, 3]. In 2018, Hardy and Leung described a fistula as the primary presentation of a high-grade endometrioid adenocarcinoma [8]. Risk factors include multiple abdominal surgeries, corporeal hysterotomy, and presence of a foreign body (18), use of drains, or incomplete closure of a uterine wound following a C-section.

The diagnosis of UCF can be challenging. The time between surgery and the diagnosis of UCF ranges from 15 days to 11 years [1]. Whereas a number of reported cases describe bleeding through the C-section scar as a pathognomonic symptom, others mention menstrual blood loss through the fistula, abdominal wall discharge, or undefined abdominal pain [1]. The differential diagnosis includes intra-abdominal infections or tumors. Persistent abdominal pain and significant weight loss should lead to imaging to rule out pelvic tumors, an intra-abdominal abscess, or a fistula. Multiple diagnostic imaging techniques are available: ultrasonography, tomography, or MRI. Others described diagnosis tools including hysterosalpingography and hysteroscopy [1].

The prevalence of infection after hysteroscopy varies between 0.18 and 1.5% [9]. Currently, guidelines do not recommend the use of prophylactic antibiotics for hysteroscopy [10, 11]. Yet limited scientific evidence supports this recommendation. A meta-analysis of 5 studies [12] shows that antibiotics do not reduce the rate of fever, endometritis, or PID after hysteroscopy [12]. However, this study has some limitations. A hysteroscopy performed after chorioamnionitis or PID is likely associated with a greater infectious risk than a hysteroscopy in a patient with no infection history. The risk of infection is also higher after a prolonged operative hysteroscopy, especially after repeated insertion and removal of the hysteroscope through the cervix [13]. Comorbidities such as immunosuppression, connective tissue disorders, heart disease, mitral valve prolapse, history of PID or pelvic abscess, tubal dilation, and endometriosis were reported as risk factors [12, 14, 15]. Screening with a vaginal swab culture and/or prophylactic antibiotics should be considered for high-risk patients, as hysteroscopy can lead to serious complications such as UCF.

The treatment of UCF includes hormonal therapy and surgical excision of the fistulous tract with or without hysterectomy. Surgery is frequently the primary treatment. Medical treatment using the GnRH agonist for 6 months has been described in two cases [16, 17]. For young patients with incomplete family planning, conservative treatment, including resection of the fistula tract and closure of the uterus, should be considered with a risk of recurrence after conservative treatment [18]. A successful pregnancy and delivery were described in 2019 after the excision of a postmyomectomy uterocutaneous fistula tract [19]. After explaining the different available treatments, the patient refused the conservative treatment. After months of persistent symptoms and medical wandering, the patient was adamant for a hysterectomy.

## 4. Conclusion

Although UCFs are rare conditions, they should be included in the differential diagnosis of persisting symptoms such as metrorrhagia, purulent vaginal discharge, pelvic pain, and significant weight loss in patients with a history of uterine procedures such as C-section, curettage, hysteroscopy, or myomectomy followed or preceded by genital infections as PID or chorioamnionitis. Imaging by a CT scan is an important diagnostic tool in these clinical situations. A fast diagnostic will improve the patient's care and enable a conservative treatment in most cases. As a preventive care, screening with a vaginal swab culture and/or prophylactic antibiotics should be considered when performing a hysteroscopy in patients with recent pelvic or genital infections.

## Data Availability

The data used in the case report are included within the article.
